# Brain tumor diagnosis from MRI based on Mobilenetv2 optimized by contracted fox optimization algorithm

**DOI:** 10.1016/j.heliyon.2023.e23866

**Published:** 2023-12-19

**Authors:** Lu Xu, Morteza Mohammadi

**Affiliations:** a Department of Interventional Therapy, The Second Affiliated Hospital of Anhui Medical University, Hefei, Anhui, 230000, China; b Department of Medical Sciences, Tehran Branch, Islamic Azad University, Tehran, Iran; c College of Technical Engineering, The Islamic University, Najaf, Iraq

**Keywords:** Brain tumor, Diagnois, MRI, Mobilenetv2, Contracted fox optimization algorithm, Deep learning, Medical imaging, Figshare dataset

## Abstract

This research paper presents an innovative approach to brain tumor diagnosis using MRI scans, using the power of deep learning and metaheuristic algorithm. The study employs Mobilenetv2, a deep learning model, optimized by a novel metaheuristic known as the Contracted Fox Optimization Algorithm (MN-V2/CFO). This methodology allows for the optimal selection of Mobilenetv2 hyperparameters, enhancing the accuracy of tumor detection. The model is implemented on the Figshare dataset, a comprehensive collection of MRI scans, and its performance is validated against other processes the results are compared with some published works including Network (RN), wavelet transform, and deep learning (WT/DL), customized VGG19, and Convolutional neural network (CNN). The results of the study, highlight the superior performance of the proposed MN-V2/CFO model compared to other tactics. The recommended strategy achieves a precision of 97.68 %, an F1-score of 86.22 %, a sensitivity of 80.12 %, and an accuracy of 97.32 %. The findings validate the potential of the proposed model in revolutionizing brain tumor diagnosis, contributing to better treatment strategies, and improving patient outcomes.

## Nomenclature

**Symbol**
CFO: Contracted Fox Optimization
MN-V2: Mobilenetv2
WT/DL: Wavelet transform and deep learning
CT: computed tomography
MRI: magnetic resonance imaging
CNN: convolutional neural network
CLAHE: Contrast-limited adaptive histogram equalization
AHE: Adaptive histogram equalization
CDF: Cumulative Distribution Function
IR: Inverted Residual
SD-WC: Separable Depth-Wise Convolution
k: The intensity value
L: The number of intensity levels
i, j: The position of each tile in the image
H(k): The count of occurrences in the histogram
C'(k): The redistributed cumulative distribution function (CDF) for the intensity value k
C(k): The cumulative distribution function (CDF) for the intensity value k
M and N: The dimensions of the image (pixels)
T: The maximum allowed difference between the maximum and minimum intensity values within a tile
$H^{\prime }\left(k\right)$: The clipped histogram count for intensity value k (counts)
x: The original value of the data point
min: The minimum pixel value in the dataset
max: The maximum pixel value in the dataset
$x_{norm}$: The normalized value of the data point
$d_{i}$: The number of feature maps in the input layers
$d_{j}$: The number of feature maps in the output layers
$w_{i}$: The width of the input feature maps
$h_{i}$: The height of the input feature maps
k: The filter size
$E_{\vec{y},\vec{z}}$: The loss function of cross entropy
N: The quantity of training samples
i: The index of each training sample
$\vec{y}$: Input tensor
$\vec{z}$: one-hot-vector specifying the ground certainties for the containing network
$P\left({\vec{y}}_{i}\right)$: Function resulting in a vector with 4 dimensions with the ${\vec{y}}_{i}$ as input, chosen from every class
$WE_{\vec{y},\vec{z},\vec{w}}$: The loss function of weighted cross entropy
⨀: The multiplication of element-wise amid 2 vectors
$F_{\vec{y},\vec{z}}$: The focal function
δ: an element that decays the loss for simpler samples
${Er}_{t,i}$: The rate of class error
${TPR}_{t,i}$: The real positive rate
t: The repetition
i: Class number
ρ (y): Transformation function

## 1 Introduction

Brain tumors are abnormal formations characterized by the anomalous proliferation of cells within the cerebral region. Brain tumors can exhibit either a benign or malignant nature, with the former denoting a non-cancerous state and the latter indicating a cancerous condition. Brain tumors can elicit a range of symptoms contingent upon their specific location and size [1]. These symptoms may encompass seizures, headaches, alterations in vision or hearing, as well as cognitive or motor impairments [2]. The timely identification and precise diagnosis of brain tumors are of paramount importance to facilitate optimal treatment strategies and enhance patient prognoses [3].

The conventional approach to diagnosing brain tumors typically entails the utilization of various medical imaging modalities, including computed tomography (CT), magnetic resonance imaging (MRI), and biopsy [4]. Nevertheless, healthcare professionals may encounter difficulties and invest a significant amount of time in the process of interpreting these images. This is the point at which artificial intelligence (AI) becomes relevant [5].

Artificial intelligence (AI), particularly deep learning and metaheuristics, have recently emerged as potentially valuable methodologies for the identification and detection of brain tumors [6]. Deep learning is a specialized domain within the field of artificial intelligence that employs artificial neural networks to derive significant patterns from extensive datasets, thereby acquiring novel knowledge. Medical imaging has demonstrated notable achievements in a range of tasks, encompassing the detection and categorization of brain malignancies, among other diverse applications [7].

Deep learning models possess the capability to analyze medical images and autonomously detect characteristics that are indicative of brain tumors. These models possess the ability to acquire knowledge from a substantial dataset comprising labeled images, enabling them to effectively categorize tumors and discern between cases that are benign or malignant. Deep learning algorithms have the capability to undergo training in order to accurately identify and delineate the tumor region within brain images. This particular task holds significant importance in the context of treatment planning and the ongoing monitoring of tumor progression [6].

In contrast, metaheuristics refer to a class of optimization algorithms that draw inspiration from natural phenomena, including but not limited to evolution, swarm intelligence, and simulated annealing [8]. The utilization of these algorithms has the potential to enhance the efficiency of deep learning models in the context of brain tumor diagnosis [9]. One potential application of metaheuristics is the optimization of hyperparameters in deep learning models, which can lead to enhancements in their generalization capabilities and overall performance [10].

The incorporation of deep learning and metaheuristics in the realm of brain tumor diagnosis presents numerous benefits. Firstly, the implementation of this technology has the potential to substantially decrease the amount of time and effort needed for the manual interpretation of medical images. As a result, it can facilitate quicker and more precise diagnoses [11]. Furthermore, the utilization of AI-based methodologies can offer healthcare professionals supplementary perspectives and decision-making assistance, resulting in enhanced precision in the formulation of treatment plans [12]. Finally, the utilization of artificial intelligence (AI) has the potential to enhance the availability of high-quality healthcare in underserved regions where there may be a scarcity of specialized knowledge and skills [13].

## 2 Related works

There are different AI-based techniques for brain tumor diagnosis. Some are based on deep learning, and some are based on metaheuristics. By the way, some others benefit from the results of a combination of these two techniques.

For example, Chattopadhyay et al. [14] presented an approach for the identification of brain tumors in MRI scans by employing a convolutional neural network (CNN) in conjunction with conventional classifiers and deep learning techniques. The model was trained by the authors using a diverse set of MRI images that encompassed a range of tumor shapes, locations, intensities, and sizes. The proposed method was implemented using the TensorFlow and Keras libraries in the Python programming language. The convolutional neural network (CNN)-based model attained a remarkable accuracy of 99.74 %, surpassing the current state-of-the-art outcomes. The researchers arrived at the conclusion that their model possesses the potential to aid medical professionals in accurately identifying brain tumors, thereby expediting the treatment process.

Mohan et al. [15] introduced a novel automated model based on deep learning techniques for the detection and classification of brain tumors utilizing magnetic resonance imaging (MRI) data. The proposed framework encompassed several key components, namely preprocessing, segmentation, feature extraction, and classification. The utilization of adaptive fuzzy filtering served as a preliminary method to diminish noise and enhance the quality of MRI scans. The utilization of chicken swarm optimization was employed for the purpose of segmenting MRI images and identifying regions within the brain that had incurred damage. The utilization of a Residual Network involved the integration of manually engineered features with deep features in order to generate a coherent set of feature vectors. Based on simulations conducted on the BRATS 2015 dataset, it was determined that the DLBTDC-MRI method exhibited superiority over other contemporary procedures in various aspects. Nevertheless, it was imperative to acknowledge the constraints of this study, which encompassed the necessity for additional verification on more extensive datasets and the possibility of partiality within the dataset employed for training the model.

Zhu et al. [16] introduced a methodology for segmenting brain tumors by utilizing multimodal magnetic resonance imaging (MRI) scans that incorporate various imaging parameters. The segmentation accuracy was achieved by integrating deep semantic features with edge information in the proposed method. The method proposed in this study comprised three distinct modules, namely semantic segmentation, edge detection, and feature fusion. The Swin Transformer was utilized by the researchers to extract semantic features. In addition, the edge detection module was constructed using CNNs. To further enhance the features, a novel edge spatial attention block (ESAB) was introduced. To integrate different features, the researchers employed a multi-feature inference block (MFIB) that utilizes graph convolution. This MFIB is responsible for merging the extracted semantic and edge features. The findings indicated that the method proposed in this study exhibited superior performance compared to various contemporary brain tumor segmentation methods as evaluated on the BraTS benchmarks.

Narmatha et al. [17] presented a novel approach, namely a hybrid fuzzy brain-storm optimization algorithm for the purpose of classifying brain tumor MRI images. A brain tumor was a debilitating condition of the nervous system that results in substantial health impairment and ultimately mortality. Magnetic resonance imaging (MRI) is a highly prevalent medical imaging modality employed for the detection and characterization of brain tumors. The process of segmenting and classifying brain tumors was a complex undertaking. The FBSO algorithm, as proposed, exhibited high efficiency and robustness. Its primary advantage lied in significantly reducing the duration of the optimization algorithm's segmentation process. Furthermore, it achieved a commendable level of accuracy, with a precision of 94.77 %, a sensitivity of 95.77 %, and an F1 score of 95.42 %.

Irmak et al. [18] aimed to develop a fully automated method for the multi-classification of brain tumors based on deep learning using a convolutional neural network (CNN). Three distinct convolutional neural network (CNN) architectures were put forth to address three separate classification tasks. The initial convolutional neural network (CNN) model demonstrated a remarkable accuracy rate of 99.33 % in the detection of brain tumors. The second convolutional neural network (CNN) model achieved a classification accuracy of 92.66 % in categorizing brain tumors into five distinct types. The third convolutional neural network (CNN) model achieved a classification accuracy of 98.14 % in categorizing brain tumors into three distinct grades. The CNN models that were suggested were evaluated against other well-known state-of-the-art CNN models and achieved favorable classification outcomes when applied to extensive clinical datasets that are accessible to the public. The utilization of the proposed convolutional neural network (CNN) models has the potential to support medical professionals, such as physicians and radiologists, in the validation of their initial screening for the multi-classification of brain tumors. Nevertheless, it is imperative to acknowledge the constraints of this study, which necessitate additional verification on more extensive datasets and the possibility of partiality in the dataset employed to train the models.

## 3 Motivation and contributions

Despite the advancements in medical imaging, there is a lack of effective automated methodologies for accurate brain tumor diagnosis. The current methods are not efficient in pinpointing the exact location and size of the tumor.

This paper is motivated by the need to improve the accuracy and efficiency of brain tumor diagnosis. The study contributes to the field by introducing an automated methodology based on deep learning and optimization. The use of Mobilenetv2, optimized by the Contracted Fox Optimization Algorithm, presents a promising solution to the identified research gap. The successful implementation of this model on the Figshare dataset further validates its potential in revolutionizing brain tumor diagnosis.

In this framework, the study aims to explore the potential of utilizing deep learning and optimization techniques for brain tumor diagnosis using MRI scans. The researchers developed a methodology that combines Mobilenetv2, a powerful deep learning model, with the Contracted Fox Optimization Algorithm (CFOA), a metaheuristic optimization algorithm.

The goal of employing Mobilenetv2 is to effectively extract relevant features from MRI scans, enabling accurate identification and characterization of brain tumors. Meanwhile, CFOA optimizes the hyperparameters of Mobilenetv2 to enhance its performance and overall diagnostic accuracy. This study builds upon previous research in the field of medical imaging and brain tumor diagnosis, with an emphasis on the potential benefits of automated deep-learning algorithms. The researchers seek to improve the precision and efficiency of brain tumor diagnosis while reducing reliance on subjective human interpretation. By showcasing the effectiveness of their methodology on the Figshare dataset, which includes a comprehensive collection of MRI scans, the researchers demonstrate the real-world applicability and potential impact of their work.

The study presented in the research paper introduces several novel aspects in the field of brain tumor diagnosis using MRI scans hat are given below.

− Integration of Deep Learning and Metaheuristic Optimization: The study combines the power of deep learning with a novel metaheuristic optimization algorithm called the Contracted Fox Optimization Algorithm (CFOA). This integration allows for the optimal selection of hyperparameters for the Mobilenetv2 deep learning model, enhancing its performance in tumor detection.
− Use of Mobilenetv2: The study utilizes Mobilenetv2, a deep learning model specifically designed for mobile and embedded vision applications. By employing Mobilenetv2, the researchers aim to effectively extract relevant features from MRI scans, enabling accurate identification and characterization of brain tumors.
− Application of the Contracted Fox Optimization Algorithm (CFOA): The researchers optimize the hyperparameters of Mobilenetv2 using the CFOA. The CFOA is a metaheuristic optimization algorithm that aids in finding the best set of hyperparameters for the deep learning model, thereby improving its overall diagnostic accuracy.
− Validation on the Figshare Dataset: The researchers implement their methodology on the Figshare dataset, which is a comprehensive collection of MRI scans. By validating their approach to this dataset, the researchers demonstrate the real-world applicability and potential impact of their work.

In general, the novelty of this study lies in the integration of deep learning and metaheuristic optimization, the use of Mobilenetv2, the application of the Contracted Fox Optimization Algorithm, and the validation of a comprehensive dataset. These novel aspects contribute to the advancement of brain tumor diagnosis and have the potential to improve the precision and efficiency of this critical medical task.

## 4 Image preprocessing

In the diagnostic process of brain tumor detection through medical imaging techniques such as magnetic resonance imaging (MRI), image preprocessing assumes a crucial role. Image preprocessing involves a sequence of operations performed on raw image data with the aim of enhancing image quality, reducing noise, and extracting significant features to facilitate accurate interpretation and analysis by medical experts.

### 4.1 Image contrast enhancement

The utilization of adaptive contrast enhancement is a prevalent method within the field of image processing, aimed at augmenting the contrast and improving the visibility of distinct features present in an image. The objective of this approach is to enhance the interpretability and visual fidelity of the image through the modification of pixel intensities, taking into account local characteristics. In contrast to conventional methods of enhancing contrast, which uniformly apply a predetermined enhancement strategy to the entire image, adaptive contrast enhancement techniques consider the specific characteristics of local regions within the image and adapt the enhancement process accordingly. The algorithm examines the pixel intensities within a given locality and applies adaptive modifications to enhance the contrast in various regions of the image.

The fundamental concept underlying adaptive contrast enhancement involves the expansion or stretching of the intensity range within individual local regions, while simultaneously maintaining the overall global characteristics of the image. This process guarantees the appropriate enhancement of both the dark and bright regions, resulting in the accentuation of details and the enhancement of visibility for significant features.

Contrast-limited adaptive histogram equalization (CLAHE) is a variant of the adaptive histogram equalization (AHE) technique designed to mitigate the issue of amplifying noise and local image artifacts, which is inherent in AHE. The Contrast Limited Adaptive Histogram Equalization (CLAHE) technique is frequently employed in the field of medical imaging, particularly in the analysis of brain tumor MRI images. Its primary purpose is to augment the detectability of intricate features within the images, thereby enhancing the overall interpretation of the visual data.

CLAHE, or Contrast Limited Adaptive Histogram Equalization, can be utilized in the preprocessing stage of brain tumor MRI images to augment the contrast of tumor regions and emphasize significant characteristics. The mathematical steps for the CLAHE are explained in the following.

#### 4.1.1 Image division

Consider an initial image, denoted as I, with dimensions M×N. This image can be represented as a matrix I(i,j), where i varies from 1 to M and j varies from 1 to N. The image is partitioned into tiles, either non-overlapping or overlapping, which are represented as T(i,j) and have dimensions H×W pixels.

#### 4.1.2 Histogram calculation

The histogram count for each possible intensity value k (ranging from 0 to L−1, where L represents the number of intensity levels) is computed for every tile T(i,j). The variable H(k) denotes the count of occurrences in the histogram for a given intensity value k. As a consequence, an array H(k) representing a histogram is generated for each tile.

#### 4.1.3 Cumulative distribution function (CDF) calculation

Evaluate the cumulative distribution function (CDF), C(k) from the histogram H(k). The CDF represents the accumulated probability of the intensity values up to k and is calculated as equation (1) [19]:

(1) C(k)=∑H(j)

where, j=0,1,…,k.

#### 4.1.4 Contrast enhancement

The process of contrast enhancement entails the redistribution and clipping of the cumulative distribution function.

a) Redistribution:

The cumulative distribution function is modified in order to evenly distribute the cumulative probability among all potential intensity values. The aforementioned task is accomplished by employing the subsequent mathematical equation (2) [20].

(2) $$C^{\prime }\left(k\right)=\left(L-1\right)\times \frac{C\left(k\right)}{MN}$$

here, symbol C′(k) denotes the redistributed cumulative distribution function (CDF) for the intensity value k.

b) Clipping

To prevent over-amplification of noise, we apply a contrast limit to the redistributed CDF ($C^{\prime }\left(k\right)$). If the difference between the maximum and minimum intensity values within a tile exceeds the contrast limit (T), we clip the histogram as follows [equation (3)] [20]:

(3) $$H^{\prime }\left(k\right)=\min\left(H\left(k\right),T\right)$$

where, $H^{\prime }\left(k\right)$ represents the clipped histogram count for intensity value k.

#### 4.1.5 Reconstructing the image

**Fig. 1:**
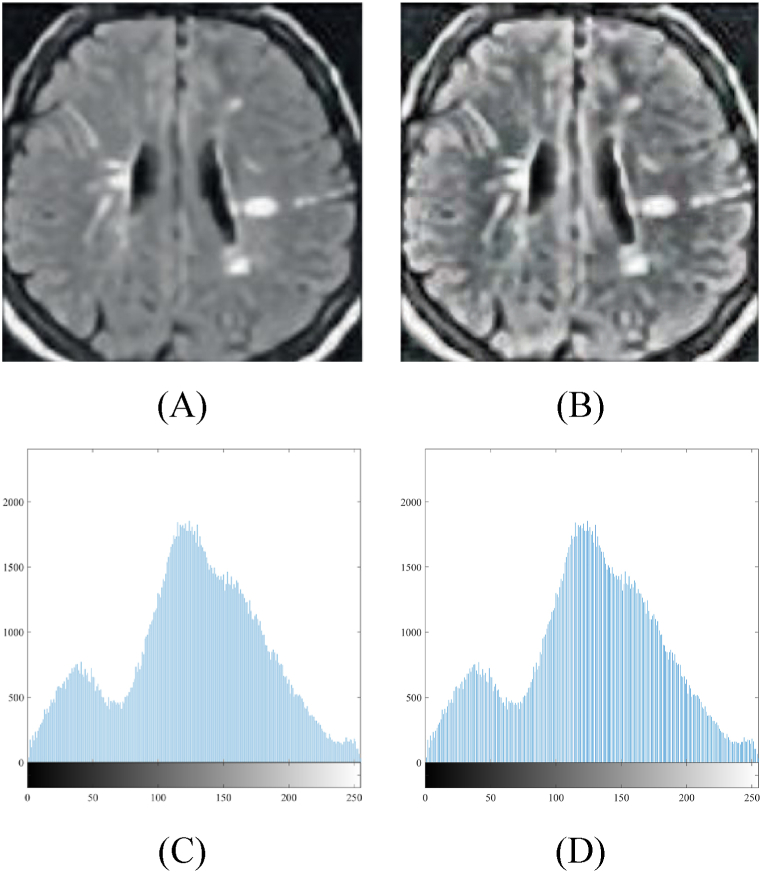
CLAHE applied to Brain tumor MRI: (A) input image; (B) image after CLAHE; (C) histogram of (A); (D) histogram of (B).

Following the application of contrast enhancement to individual tiles, the tiles are subsequently amalgamated to restore the final image. The selection of the merging technique is contingent upon the specific implementation. Fig. (1) illustrates the image contrast enhancement of a sample brain tumor MRI. The figure comprises four components: (A) the initial input image, (B) the image subsequent to enhancement, (C) the histogram corresponding to the original image, and (D) the histogram corresponding to the enhanced image.

Through a comparative analysis of the initial input image (A) and the subsequently enhanced image (B), it becomes evident that there is a notable enhancement in contrast and an augmentation of image details. The histograms depicted in figures (C) and (D) offer a graphical depiction of the alterations in intensity distribution prior to and following the implementation of Contrast Limited Adaptive Histogram Equalization (CLAHE), respectively. The efficacy of Contrast Limited Adaptive Histogram Equalization (CLAHE) in enhancing the visibility and quality of magnetic resonance imaging (MRI) scans of brain tumors is illustrated by the improved image and its corresponding histogram.

### 4.2 Min-max normalization

Min-max normalization, alternatively referred to as feature scaling or data normalization, is a method employed to adjust the scale of numerical data to a predetermined range. The primary objective of normalization is to standardize the scale of all features or variables within a dataset, thereby facilitating improved analysis and modeling capabilities. The normalization process typically involves scaling data to a range of [0, 1], although it is also possible to customize the range according to specific requirements.

The utilization of min-max normalization in the context of brain tumor diagnosis through magnetic resonance imaging (MRI) involves the process of normalizing the pixel intensities of the MRI images. The utilization of this normalization procedure facilitates a uniform and standardized examination of the images, thereby contributing to the precise identification and description of brain tumors. The min-max normalization process involves the following steps.

#### 4.2.1 Identify the minimum and maximum values

Determine the minimum (min) and maximum (max) values of the variable being normalized in the dataset.

#### 4.2.2 Define the desired range

Establish the intended interval for scaling the variable. This is commonly performed in order to establish a correspondence between the variable values and the interval [0, 1].

#### 4.2.3 Calculate the normalized value

To obtain the normalized value ($x_{norm}$) for each data point in the variable, the following formula is applied [equation (4)] [21]:

(4) $$x_{norm}=\frac{\left(x-\min\right)}{\max-\min}$$

here, x represents the original value of the data point, and min and max determine the minimum and the maximum pixel values.

#### 4.2.4 Apply normalization to the entire dataset

**Fig. 2:**
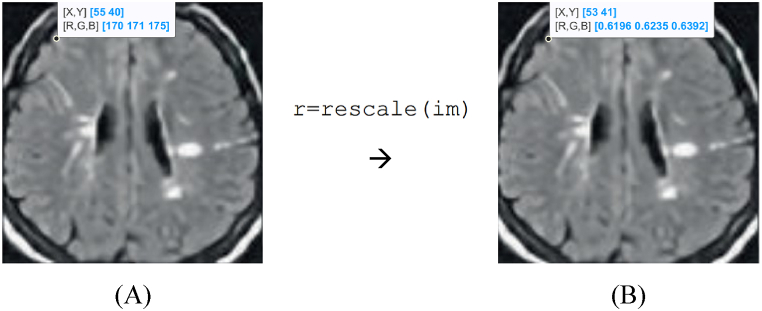
Effect of min-max normalization on brain MRI images: (A) original image, and (B) image after min-max normalization.

Repeat the normalization process for all data points in the variable or dataset. Upon the implementation of min-max normalization, the values of the variable will undergo a transformation that conforms to the predetermined range. The variable's minimum value will be assigned a mapping of 0, the maximum value will be assigned a mapping of 1, and all other values are scaled proportionally between these two extremes. Fig. (2) illustrates the effect of min-max normalization on brain MRI images: (A) original image, and (B) image after min-max normalization.

The procedure of intensity normalization entails the rescaling of intensity values to fit within the interval of [0,1]. Afterward, the intensity values that have been resized are modified to a dimension of 227 × 227 × 3 before being passed on to the Mobilenetv2 architecture. The preprocessing steps are of utmost importance in facilitating the training of networks as they improve the efficiency of the learning process and tackle potential challenges related to memory.

Min-max normalization in brain tumor diagnosis offers several advantages, including consistent pixel intensities, enhanced feature detection, quantitative analysis, compatibility with machine learning algorithms, and improved interoperability. By scaling pixel intensities to a common range, it ensures consistent pixel intensities across different images, enhancing feature detection, enabling quantitative assessment, and supporting machine learning algorithms. This results in more accurate and reliable brain tumor diagnosis, aiding in treatment planning and patient management. Overall, using min-max normalization in MRI brain tumor diagnosis improves interpretability, feature detection, quantitative assessment, and data interoperability, ultimately contributing to more accurate and reliable diagnosis.

#### 4.2.5 Data augmentation

Data augmentation is a widely employed method in the fields of machine learning and computer vision, encompassing various tasks such as medical image analysis, specifically in the domain of brain tumor diagnosis. The primary objective of this process is to artificially augment the scale and diversity of the training dataset through the application of different transformations to the pre-existing images. The utilization of data augmentation is motivated by the objective of enhancing the performance and generalizability of the machine learning model or algorithm. The introduction of variations in the training data enhances the model's robustness and its ability to effectively handle diverse scenarios and variations that may arise during real-world testing. Diverse image appearances can arise in medical imaging due to a combination of patient characteristics, imaging protocols, and scanner variations, making it particularly crucial to address this issue.

The present study employed data augmentation techniques to augment the dataset for the purpose of enhancing brain tumor diagnosis. The aforementioned techniques encompassed the application of diverse transformations to brain MRI images. The study provides a comprehensive overview of the augmentation techniques employed, which are detailed in the following.

A. Random rotation: This technique introduces random rotations to the MRI images. The lower and upper values represent the degrees of rotation, which range from −7 to 7°. By applying random rotations, the algorithm can learn to detect tumors from different orientations.
B. Random X and Y-Shear: X and Y-shear transformations introduce shearing effects to the MRI images. The lower and upper values indicate the maximum shearing amounts allowed along the X and Y axes. In this study, the range for both X and Y-shear is −0.03 to 0.03. Shearing can help simulate variations in image appearance due to patient positioning or scanner misalignment.
C. Random X and Y-reflection: This technique involves randomly reflecting the MRI images along the X and Y axes. The presence of a dash indicates that reflection was not utilized in this study. Reflection can provide additional variability to the dataset by simulating flipped orientations or anatomical differences.
D. Random X and Y-Translation: X and Y translation techniques move the MRI images horizontally and vertically, respectively. The lower and upper values determine the maximum translation allowed in pixels. In this study, the range for both X and Y-translation is −35 to 35 pixels. Translation can simulate slight shifts in patient positioning or scanner alignment.
E. Random X and Y-scale: X and Y scaling apply random scaling transformations to the MRI images. The lower and upper values specify the scaling range. In this study, the range for both X and Y-scale is 0.4–3. Scaling can simulate variations in image resolution, zooming effects, or differences in patient anatomy size.

**Fig. 3:**
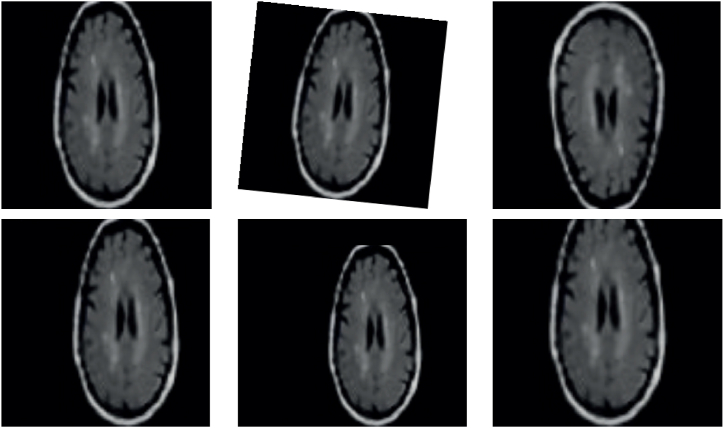
Collection of sample data augmented for the brain MRI.

This technique enhances the diversity of the dataset and enhances the robustness and generalizability of the brain tumor diagnosis model through the implementation of these data augmentation techniques. The utilization of an augmented dataset, which includes images that have undergone transformations, enables the algorithm to acquire knowledge from a broader spectrum of variations that are frequently encountered in real-world situations. This augmentation process enhances the algorithm's capacity to effectively detect and classify brain tumors with a higher degree of accuracy. Fig. (3) displays a collection of sample images that demonstrate the utilization of data augmentation techniques.

Fig. (3) presents a compilation of illustrative images that demonstrate the practical implementation of various data augmentation techniques on a representative image. As previously stated, the methods incorporated in this collection consist of X and Y scaling, shearing, reflection, translation, and rotation by a designated angle. The purpose of utilizing these augmentations is to introduce variability and enhance the diversity of the dataset, thereby enhancing the model's ability to generalize and accurately identify patterns across different perspectives and circumstances. By incorporating these modifications, the model acquires improved resilience and adaptability in diverse scenarios, leading to increased effectiveness and reliability when utilized in real-world applications.

## 5 Contracted fox optimization algorithm

### 5.1 Background

At first, the population is initialized by FOX called X matrix. The position of red foxes is shown by X. in each iteration, by the use of standard objective function, each search agent fitness is computed. The cost value of every search agent is juxtaposed to the cost of other agents to explore BestFitness and best position (BestX). The fitness of the current row $\left(fitness_{i+1}\right)$ and fitness of the preceding row $\left(fitness_{i}\right)$ over iterations are compared and the results of BestFitness and BestX are reported.

Furthermore, a condition must be utilized with a parameter that is random to balance the phases of exploitation and exploration. Regarding the quantity of iteration, the phases are divided equally by this parameter. The remaining half of the iterations are utilized for exploitation, with approximately half being used for exploration. by the use of a random parameter which is called r. Regarding the avoidance of local optima and balancing, the suggested algorithms are really necessary. Hence, in order to allocate equal chances to both exploitation and exploration, a statement of condition is utilized. In order to dilute search efficiency in accordance with the BestX, the parameter a is used. The value is reduced each time the process of iteration is conducted. Thus, in each iteration, the agent chases the prey better than the previous ones. The search agents attempt to avoid local optima due to the effects of the value of fitness. The reason for this is that if novel positions are not altered, in order to allow other phases to be activated, the phase of exploration is deactivated. Two phases of exploration and exploitation are explained thoroughly in the following.

#### 5.1.1 Exploitation

In this phase, prey might be killed due to a condition as mentioned before. The range [0, 1] encompasses the value of the random variable called p. It is noteworthy that there is a need to find a novel position for the red fox if the p is larger than 0.18. The value of jumping, the distance of the fox from the prey, and the sound travel time which are, in turn, called Jumpit, $Dist\_Fox\_Prey_{it}$, and $Dist\_S\_T_{it}$, must be computed to discover a novel position. Eventually, there is a number, at random, for the sound travel time $Time\_S\_T_{it}$ that ranges from 0 to 1 and has been generated. By multiplying time sound travels time $Time\_S\_T_{it}$ with the speed of sound in the air Sp_S, the sound distance can be found [equation (5)] [22]:

(5) $$Dist_{S_{T_{it}}}=Sp_{S}\times Times\_S\_T_{it}$$

$Time\_S\_T_{it}$ is a random number between [0, 1], and the sound speed in the medium Sp_S is 343 in the air. The number of iterations is shown by it, and it ranges from 1 to 500. Another formula has been taken to discover Sp_S with respect to the best location which has been discovered up to now by dividing sound time travel between prey and fox. The best and random agents are, in turn, $BestPoistion_{it}$ and $Time\_S\_T_{it}$. The way of discovering the speed of the sound Sp_S is illustrated in equation (6) [22].

(6) $$Sp\_S=\frac{BestPosition_{it}}{Time\_S\_T_{it}}$$

**Fig. 4:**
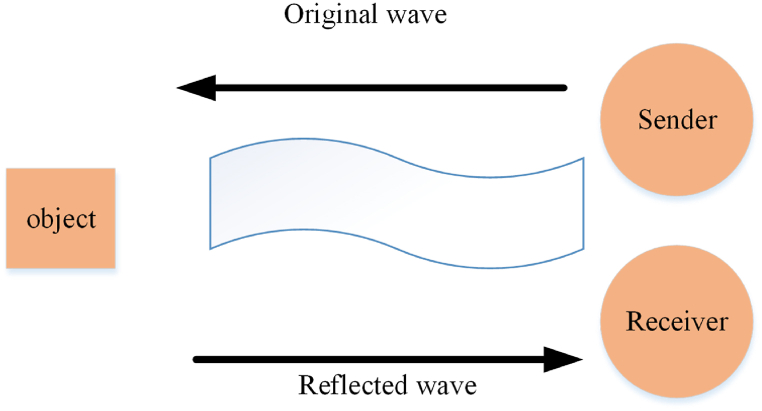
Calculating distance by ultrasound sensor.

Eq. (1) is used in order to find the distance that sound travels. Eventually, If $Dist\_S\_T_{it}$ gets halved, the distance of the fox from the target, $Dist\_Fox\_Prey_{it}$, can be computed [22]. In Fig. 2, it is represented how the sensor sends a sound wave to an object and after a while how it receives the signal again. So, the sound travel distance is multiplied by the sensor at the rate of 0.5. or 12. Employing the first or second one does not make any difference since half of the signal is desired. In order to find the distance from Fig. (4), strategies of objects and sensors are used which are explained in more detail in equation (7) [22].

(7) $$Dist\_Fox\_{Prey}_{it}=Dist\_S\_T_{it}\times 0.5$$

When the distance between the prey and the red fox is found, it is time for the red fox to discover a new position so that it is required to jump to have the target. So, the red fox has to compute the amount of height it has to jump which is known to be $jump_{it}$. Moreover, the equation below calculates $ump_{it}$ .

(8) $$Jump_{it}=0.5\times 9.81\times t^{2}$$

t is the average time for sound to travel and it is squared because of the up-and-down steps during steps; moreover, 9.81 is the acceleration caused by gravity. By dividing the sum of the $Time\_S\_T_{it}$ to dimensions, the value of time transition tt can be computed. The calculation of MinT and tt is illustrated by eq. (7). By dividing tt by 2, the mean time t can be calculated. Because the value of jump goes up and down two various times, the gravity and the meantime are multiplied by 0.5. afterward, the value of jump is multiplied by $c_{1}$ and $Dist\_Fox\_Prey_{it}$. Each time the fox jumps in the direction of the northeast, the variable $c_{1}$ is in the range of [0, 0.18]. The novel position of the fox is calculated by the formulation below if the p is larger than 0.18 [equation (9)] [22].

(9) $$X_{it+1}=Dist\_Fox\_Prey_{it}\times Jump_{it}\times c_{1}$$

In order to find a new position for the fox, Eq. (5) and Eq. (6) have been utilized. Due to using p, only one of these equations can be performed in each iteration. But, there is merely one variation which is in the second section of the p in the above formulation. If p≤0.18, the above formulation is multiplied by $c_{2}$ not $c_{1}$. It is noteworthy that the novel location must be computed by the above formulation if p>0.18. Furthermore, [(equation (10)] the novel location must be calculated by the equation above if p<0.18. $c_{2}$ is in the range of [0.19, 1].

(10) $$X_{it+1}=Dist\_Fox\_Prey_{it}\times Jump_{it}\times c_{2}$$

0.82 and 0.18 are, in turn, the values of $c_{2}$ and $c_{1}$. The movement of a fox can determine the mentioned values if the jump to the northeast or other directions. So, the fox jumps to the direction of northeast if p>0.18. Eventually, $Jump_{it}$ and $Dist\_Fox\_Prey_{it}$ have been multiplied by $c_{1}$ to discover a novel location. Correspondingly, it is really likely to exploit a novel location. On the other hand, if p<0.18, the fox certainly jumps to the opposite side of the northeast. It means that is less likely to kill the prey approximately 18 %. That is why, $Jump_{it}$ and $Dist\_Fox\_Prey_{it}$ have been multiplied by $c_{2}$.

#### 5.1.2 Exploration

In this phase, the fox investigates randomly in accordance with the finest position of the fox that has been discovered up to now in order to control the random walk. There is no specific strategy for jumping in this phase since the fox must walk and search randomly to discover prey in an area that is to be searched. The variable a and a minimum time variable MinT are utilized to control the investigation, in order to make sure that the fox walks and searches randomly. Computation of the MinT and the variables are illustrated in equation. (11) and equation. (12). By finding the minimum amount of tt, MinT can be calculated [22].

(11) $$tt=\frac{sum\;\left(Tim{e_{S_{T}}}_{it}\left(i,:\right)\;\right)}{dimension},MinT=\mathrm{Min}\left(tt\right)$$

In order to calculate the average of minimum time tt, the sum of $Time\_S\_T_{it}\;\left(i,:\right)$ has been divided by problem dimensions [22].

(12) $$a=2\times \left(it-\left(\frac{1}{Max_{it}}\right)\right)$$

The maximum number of iterations is shown by $Max_{it}$. The crucial effect of a and MinT variable is that it helps the search phase to go toward the solution which is close to the finest one. For making sure that the fox walks randomly to discover the prey, rand (1, dimension) should be utilized. But, variables a and MinT have been utilized to improve the investigation ability of the fox. Balancing exploitation and exploration phases highly depends on variable r that is counted as a random number.

The finest solution $BestX_{it}$ has been discovered to have an incredible influence on the phase of exploration. The exploration strategy of the fox in investigating a novel location in the search area $X_{it+1}$ has been illustrated in Eq. (13). The formulas in this stage can be adjusted to present algorithms to improve their efficiency. They can be utilized to suggest a novel metaheuristic algorithm, as well [22].

(13) $$X_{\left(it+1\right)}=BestX_{it}\times rand\left(1,dimesnion\right)\times MinT\times a$$

When FOX is utilized to solve the multidimensional problem of space, the formulations in the phases only need to be adapted to a special problem.

In general, the first stage for FOX is that the population of the red fox should be initialized at random. After that, in order to make sure that the location of every fox has been inside the bounder of the cost function, the population is checked. Then, the fitness value of the objective function, regarding the row of population, is computed. In the end, the best position (BestX) and the BestFitness are chosen. Afterward, by contrasting the random number r, a state gets begun. It should be noted that the phase of exploitation gets activated if the stochastic number is larger or equal to 0.5.

Moreover, there is a condition in the phase of exploitation called p. The novel location should be found for the red fox if the value of p is larger than 0.18. But, the novel location must be calculated if the value of p is equal to or less than 0.18. In other situations, the phase of exploration gets activated if the r is less than 0.5. The random number, multiplying variables MinT and a, and the best position are some ways to discover the novel location. In the first iteration, the BestFitness is returned. Identical steps are taken to find the finest location and the finest fitness in the second iteration continuously after improving the population.

Considering the mathematical complication of Fox, it can be concluded that there is a time complication in O(SearchAgents×D×it) in each iteration. Here, SearchAgents is the size of the population, it refers to the iteration number, and D is the problem dimension. Hence, the FOX possesses an $O\left(n^{2}\right)$ as a complication of time. In addition, the FOX space complication can be computed considering the metrics. Therefore, for each iteration, the FOX possesses an $O\;\left(n^{2}\right)$ as a complication of space.

### 5.2 Contracted fox optimization algorithm

The need to modify an algorithm arises when there is a desire to enhance its performance, address specific limitations, or adapt it to a particular problem domain. In the case of the “Fox Optimization Algorithm”, the modification is aimed at improving its exploration and exploitation capabilities, which are crucial for effective optimization. The modification applied to the Fox Optimization Algorithm involves two key components: Lévy flight and an elimination phase.

#### 5.2.1 Lévy flight

Lévy flight is a random walk process that incorporates long jumps, allowing the algorithm to explore the search space more efficiently. By introducing Lévy flight into the Fox Optimization Algorithm it enhances the algorithm's ability to escape local optima and discover promising regions in the search space that may contain better solutions. The formulation of the Lévy flight model aims to improve the optimization of local exploration in a random walk process. This is explicated by the following formulas [equation (14) and equation (15) and equation (16)] [23]:

(14) $$\text{Lf}\left(w\right)\approx \frac{1}{w_{i|i=1,2}^{1+\xi }}$$

(15) $$w_{i}=\frac{A}{{\left|B\right|}^{1/\xi_{i}}}$$

(16) $$\sigma^{2}={\left\{\frac{\Gamma \left(1+\xi_{i}\right)}{\xi_{i}\Gamma \left(\left(1+\xi_{i}\right)/2\right)}\frac{\sin\;\left(\pi \xi_{i}/2\right)}{2^{\left(1+\xi_{i}\right)/2}}\right\}}^{\frac{2}{\xi }}$$

In this framework, variables A and B are denoted as two quantities that have been assigned a mean value of 0 and a variance of $\sigma^{2}$. The function Γ(.) represents the Gamma function, while the parameter w defines the size step. The variable ξ, which lies within the range [0,2], specifies the Lévy index, subject to a certain condition. In this background, the symbol ξ represents the set 3/2 [24]. The stochastic variable r can be updated according to the principles of the Lévy flight process. They can be utilized to suggest a novel metaheuristic algorithm, as well [equation (17)].

(17) $$X_{\left(it+1\right)}=BestX_{it}\times Lf\left(\delta \right)\times MinT\times a$$

where, Lf(δ) specifies the levy flight value.

#### 5.2.2 Elimination phase

The elimination phase is a mechanism that removes poor solutions from the population, improving the overall quality of the solutions. In this phase, the algorithm evaluates the fitness of each solution and eliminates a certain percentage of the worst-performing solutions. This helps maintain a diverse population and prevents the algorithm from getting stuck in suboptimal regions. The primary aim of the elimination phase is to eliminate candidate solutions from the population that is expected to be suboptimal, thereby facilitating the achievement of the elimination phase's purpose. The algorithm demonstrates the ability to allocate its computational resources towards particular regions within the solution space that present greater potential.

This is achieved by first discarding solutions with lower potential and then proceeding to explore the following areas of the solution space. This methodology assists in reducing the likelihood of becoming fixated on a solution that is only optimal within a specific context. This phase also facilitates the promotion of diversity within the population, thus allowing the algorithm to systematically explore different regions of the search space and ultimately converge toward optimal solutions.

The parameter mentioned above is integrated into the traditional FOA framework, and its calculation depends on the number of iterations. The aforementioned modifications are implemented by taking into account three distinct factors, specifically ep, et, and th.

Given a parameter value of 40 for ep, the elimination phase will occur once every 40 iterations. The parameter that was previously discussed has been integrated into the conventional FOA model. The value of the variable is ascertained by computing the ratio of the initial population size. Based on the provided parameters, which include a population size of 60 individuals and a value of thirty for et, it can be deduced that in each iteration of the elimination process, a total of thirty candidates with lower cost values are eliminated. The removal of candidates has led to the replacement of random candidates within the revised exploration domain.

**Fig. 5:**
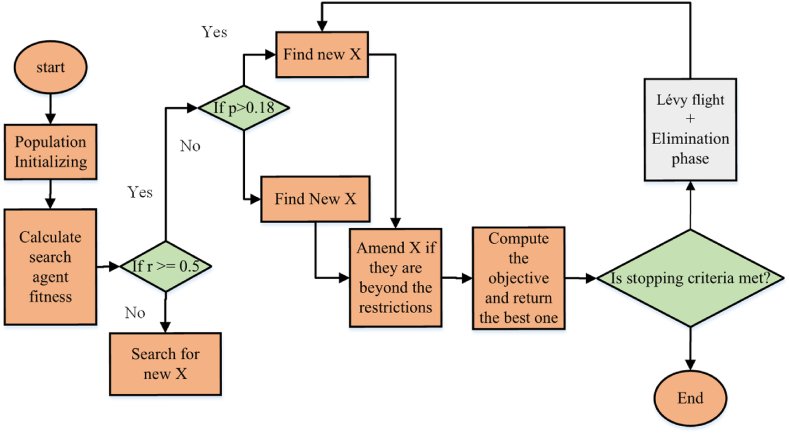
Details of the fox.

In the chosen domain of study, the term “th” denotes the proportion of either the utmost or minimum attainable magnitude. The parameter Th has the potential to assume a value of 80, despite the initial range for parameter exploration being limited to a maximum range of −1 to 1. This denotes an exceptional instance of the situation under consideration. Throughout each iteration of the elimination procedure, a comprehensive evaluation is conducted on the particle's parameters to ascertain if they exceed 60 % of the absolute magnitude of the solution space. Upon the fulfillment of the necessary prerequisite, an enhancement will be incorporated into the realm of investigation pertaining to the parameter under consideration. Fig. (5) demonstrate the details of the FOX.

The inclusion of Lévy flight in the algorithm improves exploration, enabling the algorithm to find global optima more extensively. This overcomes the challenge of being trapped in local optima. The elimination phase focuses on exploiting promising solutions, improving convergence speed and final solutions quality. Additionally, the elimination phase maintains a diverse population by removing poor solutions, promoting better exploration of the search space, and preventing premature convergence, resulting in a more robust and reliable optimization process. By integrating the Lévy flight mechanism and the elimination phase into the proposed algorithm, the revised version demonstrates the ability to effectively mitigate the issue of local optima, expedite the convergence rate, and augment the overall quality of the acquired solutions. The aforementioned modifications enhance the algorithm's effectiveness and efficiency in addressing optimization problems.

### 5.3 CFOA verification

To validate the CFOA, a comprehensive evaluation is conducted on standard benchmark functions. The validation process involves comparing the results of the modified algorithm with five different state-of-the-art methods, including Pelican Optimization Algorithm (POA) [25], Tunicate Swarm Algorithm (TSA) [26], Gravitational Search Algorithm (GSA) [27], Owl Search Algorithm (OSA) [28], and Pigeon-inspired Optimization Algorithm (PIO) [29]. The parameter setting of the algorithms are selected based on rials and errors. However, the population size and the iteration of all algorithms are set to 60 and 200, respectively to provide a fair comparison. The parameter setting of the algorithms are given below.

- Pelican Optimization Algorithm (POA):

I: The number of iterations or generations in the algorithm is set to 2.

R: The rate of randomization, which controls the exploration-exploitation trade-off, is set to 0.4.

T: The number of pelicans or search agents in the algorithm is set to 200.

- Tunicate Swarm Algorithm (TSA):

Search agents: The number of search agents or tunicates in the algorithm is set to 60.

$P_{\min}$: The minimum number of particles in a swarm is set to 1.

$P_{\max}$: The maximum number of particles in a swarm is set to 4.

Number of generations: The number of generations or iterations in the algorithm is set to 200.

- Gravitational Search Algorithm (GSA):

Search agents: The number of search agents or particles in the algorithm is set to 60.

Gravitational constant: The gravitational constant, which determines the strength of the gravitational force, is set to 100.

Alpha coefficient: The alpha coefficient, which controls the influence of the gravitational force, is set to 20.

Number of generations: The number of generations or iterations in the algorithm is set to 200.

- Owl Search Algorithm (OSA):

$T_{dead}$: The maximum number of iterations an owl can stay in the same position without improvement is set to 15.

|P|: The number of owls or search agents in the algorithm is set to 12.

$Acc_{low}$: The lower acceleration coefficient, which controls the exploration phase, is set to 0.1.

$Acc_{high}$: The higher acceleration coefficient, which controls the exploitation phase, is set to 1.

- Pigeon-inspired Optimization Algorithm (PIO):

Number of Pigeons: The number of pigeons or search agents in the algorithm is set to 60.

Space dimension: The dimensionality of the search space is set to 15.

Map and compass factor: The factor that determines the influence of the map and compass mechanism is set to 0.2.

Map and compass operation limit: The maximum number of iterations for the map and compass mechanism is set to 120.

Landmark operation limit: The maximum number of iterations for the landmark mechanism is set to 150.

Inertia factor (w): The inertia factor, which controls the impact of the previous velocity, is set to 1.

Self-confidence factor ($c_{1}$): The self-confidence factor, which controls the impact of the personal best position, is set to 1.4.

Swarm confidence factor ($c_{2}$): The swarm confidence factor, which controls the impact of the global best position, is set to 1.4.

These parameter values are specific to each algorithm and have been chosen based on the characteristics and behavior of the algorithms. The values are typically determined through experimentation and fine-tuning to achieve optimal performance for different optimization problems.

The validation is performed on a set of standard benchmark functions from the “CEC-BC-2017 test suite”. To ensure reliable results, each algorithm, including the proposed modified algorithm, is run 25 times on all test functions. The number of runs is chosen to account for the stochastic nature of metaheuristic algorithms and to obtain statistically significant results. The assessment of algorithmic efficiency in the current investigation relies on three metrics: the Best, Mean, and standard deviation (StD) values of the analyzed functions. The comparison result of the CFOA and the other algorithms under study is presented in Table 1.

**Table 1:** Comparison results of the CFOA and the other algorithms under study.

Function	indicator	POA [25]	TSA [26]	GSA [27]	OSA [28]	PIO [29]	CFOA
F1	Best	1.71	2.70	2.04	76.46	2.61	1.99
	Mean	12.83	7.83	11.59	197.82	12.86	11.54
	StD	6.47	5.25	7.94	114.04	13.59	15.07
F2	Best	4.17	0.07	2.58	6.93	3.92	6.05
	Mean	45.97	0.53	52.90	62.15	63.47	61.24
	StD	32.76	0.23	52.86	21.83	58.00	33.24
F3	Best	23.13	0.00	1.93	19.70	2.78	0.01
	Mean	34.62	0.00	12.23	16.84	17.48	0.04
	StD	4.13	0.00	9.40	5.38	12.64	0.01
F4	Best	4.58	0.00	5.36	5.78	8.37	0.12
	Mean	8.84	0.00	8.20	5.01	10.96	0.39
	StD	1.93	0.00	1.09	0.86	1.57	0.15
F5	Best	2.36	0.00	0.12	1.17	0.20	0.00
	Mean	3.92	0.00	1.17	4.59	1.33	0.01
	StD	1.43	0.00	1.15	0.51	1.38	0.00
F6	Best	0.12	0.00	0.86	0.16	1.17	0.00
	Mean	0.69	0.00	0.72	0.85	1.23	0.00
	StD	1.29	0.00	1.51	1.19	1.63	0.00
F7	Best	0.41	0.02	0.44	0.69	0.57	0.55
	Mean	0.92	1.24	0.75	1.15	1.32	1.84
	StD	0.20	0.15	0.18	0.20	0.24	0.24
F8	Best	7.49	10.31	12.28	7.40	13.66	12.75
	Mean	11.36	8.99	8.65	10.55	12.44	15.99
	StD	4.63	0.16	5.94	2.14	6.60	6.36
F9	Best	11.99	0.00	0.08	5.45	0.16	0.00
	Mean	26.24	0.00	1.88	25.98	2.60	0.00
	StD	6.61	0.00	0.85	7.70	1.53	0.00
F10	Best	56.33	3.50	1.79	3.99	3.47	0.16
	Mean	140.38	8.90	6.17	9.37	12.09	2.65
	StD	40.23	8.30	3.46	4.21	5.56	6.94
F11	Best	0.07	0.00	0.06	0.06	0.11	0.00
	Mean	0.08	0.00	0.38	0.16	0.40	0.01
	StD	0.06	0.00	0.13	0.03	0.14	0.00
F12	Best	0.00	0.00	0.00	0.00	0.00	0.00
	Mean	0.00	0.00	0.00	0.00	0.00	0.00
	StD	0.00	0.00	0.00	0.00	0.00	0.00

Table 1 presents the comparison results between the CFOA algorithm and other algorithms under study. The table provides the performance indicators for different functions. For function F1, the CFOA algorithm achieves a “Best” value of 1.99, while the other algorithms achieve values of 2.70, 2.04, 76.46, and 2.61, respectively. In terms of the mean value, the CFOA performs at 11.54, compared to 7.83, 11.59, 197.82, and 12.86 for the other algorithms. The standard deviation for the CFOA method is 15.07, while it is 5.25, 7.94, 114.04, and 13.59 for the other algorithms. Similar comparisons are provided for functions F2 to F12, showcasing the "Best" value, mean value, and standard deviation for each algorithm. It is important to note that these results are relative and should not be interpreted as definitive. The choice of the most suitable algorithm depends on the specific requirements and problem at hand.

## 6 MobileNetV2 (MN-V2)

### 6.1 Configuration and model

A set of MN-V2 networks have been offered in the represented paper. These proposed networks are considered a solution for the issue of colposcopy pictures’ sorting. There were some motivations that cause the architecture selection of the MN-V2. A network training on visual identification is exposed to the occurrence of overfitting on the grounds that the utilized dataset was approximately small. Whereas utilizing an expressive and smaller network i.e., the MN-V2 refuted such an effect. The MN-V2 has been a configuration that does optimize the use of memory as well as the velocity of execution at a minimum cost when it comes to the error. When the velocity of execution is high, the tuning and experimenting of the parameter become far much simpler. Whereas, in the framework of a network set, a desired quality is the small use of memory. For explaining the configuration of the MN-V2, there have been 2 vital conceptions including the Inverted Residual(IR), and Separable Depth-Wise Convolution (SD-WC). These mentioned concepts are to be described in the following.

The SD-WC has been utilized in other effective simulations, namely ShuffleNet, Xception, and MN-V2. The conventional convolution is changed into the SD-WC by 2 operations. A feature map-wise convolution has been the 1st operator; it is a distinct convolution utilized for each map of the feature. The gained maps of the feature have been piled up and processed via a point-wise convolution, the 2nd operation. This operation has been applied to the entire feature maps simultaneously and it has a 1 × 1 kernel. The picture has been processed by a conventional convolution across its channel dimensions, height, and width in the meanwhile. Simultaneously, the SD-WC does process the picture across its height and width throughout the 1st process and it does process the channel dimension of the image through the 2nd operation. The cost computed for the SD-WC and conventional convolution can be defined as below equations (18), (19) [30]:

(18) $$C_{Nor}=h_{i}.w_{i}.d_{i}.d_{j}.k^{2}$$

(19) $$C_{Sep}=h_{i}.w_{i}.d_{i}\left(d_{j}+k^{2}\right)$$

where $C_{Nor}$ and $C_{Sep}$ signify the cost associated with conventional convolution and SD-WC. The index of output and input layers are illustrated by j and i. The quantity of the input and output maps of the feature is demonstrated by $d_{i}$ and $d_{j}$. The width and height for the input maps of the feature are signified by $w_{i}$ and $h_{i}$. Finally, k is a sign of filter sizing.

The advantage of utilizing SD-WC regarding conventional convolution can be determined based on the below formula (equation (20)) [31]:

(20) $$\frac{C_{Nor}}{C_{Sep}}=\frac{d_{j}.k^{2}}{d_{j}+k^{2}}$$

In the model of ResNet, the residual and inverted blocks have been a crucial component. In these blocks, 3 convolutional operators as well as residual links and bottlenecks have been utilized. 1×1 filters are utilized in the 1st and last operators, transferring statics from the input layer to a middle one and from the middle layer to the output one.

A 3×3 filter helps the processing action in the intermediate layer. In the block of residual, there have been more channels (maps of feature) in the last and 1st convolutions in comparison with the block internal convolution. In the inverted residual, however, fewer channels have been utilized in the last and 1st convolutions in comparison with the interior convolution. In the two of them, the residual linking stands amid the 1st and last channels (maps of feature), which quantity are far less in the MN-V2 model in comparison with the model of ResNet. If multiple units have been piled up in both models, a change of smaller and larger layers has resulted. The indispensable changes occur in the residual links’ settlement, causing a merit when it comes to memory using in the configuration of MN-V2.

A widely presented application of the MN-V2 model has been utilized in this work that is from the PyTorch Hub. Details for all the MN-V2 network applications have existed in the pieces of literature, whereas their source code is published in the link of GitHub. In the following section, a fundamental alteration of structure is to be explained which has been executed to the discussed network with the purpose of adjusting it to the problem specified in this paper.

#### 6.1.1 Tuning of MobileNetV2 (MN-V2)

The network of MN-V2 is a chain of blocks classified as inverted residuals and they are piled up amid 2 convolutions, which could act as connecters changing the input to a middle layer and this one to the output layer. The ultimate output of the convolution has been crossed throughout 2 layers named global mean pooling and inference. The network of MN-V2 has been adjusted to the problem of ImageNet sorting with 1000 classes. That's why the last layer of the convolution layer has been bigger with 1280 channels (maps of feature) and one filter with 1×1 sizing.

The problem of brain tumor sorting, however, owns just 2 classes. Therefore, it can cause the employing of a minor representation of the picture which causes achieving a superior accuracy for the network irrespective of employing alone or in the set. The last layer of the convolution, next to the residual block that is last inverted, has been altered for restraining the representation sizing to output 32 or 64 channels in lieu of 1280. This layer converts to the ultimate one providing that it is utilized inside the set.

As a consequence, the obtained maps of features have been linked via those from other simulations and managed by the collective network. On the other hand, the maps of features for this layer cross over the global mean pooling and lead to a vector with 32/64 components providing that an alone model has been employed. Then this vector has been processed via an entirely linked layer for computing the outcome. For the protection of the previously trained variables, other layers remained the same on the grounds that the original model of MN-V2 model was trained on ImageNet. What is more, the entire network is fine-tuned throughout the process of training for the mentioned reason.

### 6.2 Loss function

Due to an imbalance in the dataset, the model that is trained may be biased for better recognition of definite classes to the other detriment. That occurs due to the fact that the model has encountered samples that are uneven and they can be selected from diverse classes throughout the process of training. Hence, the variables in the model have been renewed more and more in a specific direction. For retaliating such occurrences, a model has been created that has a care concentration on the detrimental classes over the process of training. This aim can be gained by utilizing the loss function. The model has been trained based on the formula of loss of cross entropy by default as illustrated below [equation (21)] [32]:

(21) $$E_{\vec{y},\vec{z}}=-\sum_{i}^{N}{\vec{z}}_{i}\;\ln\;\left(P\left({\vec{y}}_{i}\right)\right)$$

where, $E_{\vec{y},\vec{z}}$ demonstrates the loss function of cross entropy. N and i signify the training samples' quantity and index, respectively. $\vec{y}$ and $\vec{z}$ list input tensors and one-hot-vectors specifying the ground certainties for the containing network. $P\left({\vec{y}}_{i}\right)$ is a function resulting in a vector with 4 dimensions with the ${\vec{y}}_{i}$ as input chosen from every class; achieved via operating an onward pass throughout the network with ${\vec{y}}_{i}$ and employing softmax.

The loss function of weighted cross entropy does utilize a vector named $\vec{w}$ (weight vector). This vector is defined for the purpose of putting more and more significance on several classes. In the loss function of focal, the simpler instances have been decayed utilizing an element: ${\left(1-P\left({\vec{y}}_{i}\right)\right)}^{\delta }$. The formulas for these two functions have been demonstrated below [equations22 (22), (23)] [33]:

(22) $$WE_{\vec{y},\vec{z},\vec{w}}=-\sum_{i}^{N}{\vec{z}}_{i}\left[\vec{w}⨀\ln\;\left(P\left({\vec{y}}_{i}\right)\right)\right]$$

(23) $$F_{\vec{y},\vec{z}}=-\sum_{i}^{N}{\vec{z}}_{i}\left[{\left(1-P\left({\vec{y}}_{i}\right)\right)}^{\delta }⨀\ln\;\left(P\left({\vec{y}}_{i}\right)\right)\right]$$

here, ${WE}_{\vec{y},\vec{z},\vec{w}}$ signifies the loss function of weighted cross entropy. $\vec{w}$ illustrates a class weights' vector with 4 dimensions. ⨀ shows the multiplication of element-wise amid 2 vectors, the element i from the 1st and 2nd vector. This does result in a vector with the same dimensions as the product elements. Finally, $F_{\vec{y},\vec{z}}$ depicts the focal function and δ is an element that decays the loss for simpler samples.

As mentioned, the class imbalance has been counted via a mechanism in the loss function named the simple cross entropy ($E_{\vec{y},\vec{z}}$). The network no longer learns at the identical ratio from the whole sample. Whenever the possibility $P\left({\vec{y}}_{i}\right)$ tends to 1, the natural logarithm of it becomes 0($\ln\;(P\left({\vec{y}}_{i}\right)\to 0$). Thus, the learning does saturate when the model does get more and more assured.

$WE_{\vec{y},\vec{z},\vec{w}}$ (the loss function of weighted cross entropy) has been beneficial whenever several classes have been far more significant compared to the others or they require more consideration throughout training. 2 variants have been used in our experiment: the 1st one, $\vec{w}$ was remained constant considering the size of the population, and at the 2nd one, $\vec{w}$ was changed after every repetition utilizing the ratios of class error that could be calculated based on the below formula [equation (24)] [34]:

(24) $${Er}_{t,i}=1-{TPR}_{t,i}$$

where ${Er}_{t,i}$ and ${TPR}_{t,i}$ represent the rate of class error and the real positive rate, whereby t and i demonstrate the repetition and class number, respectively.

For a particular class, the error ratio has been also equivalent to the untrue negatives’ sum. For every ${Er}_{t,i}$, ρ transformation has been utilized which provides more significance to the error ratios that are upper. The weights of the class can be determined using the below formula [equation (25)] [34]:

(25) $$w_{t,i}=\frac{\rho \left({Er}_{t,i}\right)}{\sum_{j}^{4}\rho \left({Er}_{t,j}\right)}$$

When ρ (y) = y (i.e., the function of identity), there would be a direct correlation between the weights and the rates of error. What is more, when ρ (y) = $e^{y}\text{,}$ the function of softmax would result. In this work, ρ (y) was set $y^{3},e^{y},e^{10y},and\;e^{100y}$. Over every repetition, there is more emphasis on the required classes that are done by the loss function. Whenever a lengthier period was trained and the progress is slow, that would become more effective. Therefore, $10^{-5}$ was utilized as a rate of learning that has been less than $10^{-4}$ employed through normal training.

Compared to the loss function of weighted cross entropy ($WE_{\vec{y},\vec{z},\vec{w}}$), the loss function of focal ($F_{\vec{y},\vec{z}}$) concentrates more on an even granular level. In lieu of having a care focus on a definite class, $F_{\vec{y},\vec{z}}$ does utilize diverse weights for every sample of training irrespective of the belonged classification.

Optimizing MobileNetV2 using metaheuristics involves defining the objective, choosing a metaheuristic algorithm, encoding and decoding the loss function, creating a fitness function, initializing the metaheuristic algorithm, performing iterative optimization, and validating and fine-tuning the loss function. This can improve model performance, reduce overfitting, generalization, and robustness. This study used a contracted fox optimization algorithm for this purpose.

In the proposed Mobilenetv2 optimized by the Contracted Fox Optimization Algorithm (CFOA), it's important to clarify that Mobilenetv2 itself has its own set of hyperparameters, while the CFOA is responsible for optimizing these hyperparameters. The hyperparameters specific to Mobilenetv2 include as follows: Depth Multiplier, which is set to 0.5, which reduces the number of channels in each layer by half, balancing model complexity and computational cost. Input Resolution, which is set to 512 × 512. Width Multiplier which is set to 0.75 reduces the number of filters in each layer by 25 %, and the Dropout Rate which is set to 0.2 means that during training, 20 % of the input units are randomly set to 0.

## 7 Simulation results

### 7.1 Dataset description

The dataset under consideration is named “Figshare dataset”, which is publicly accessible via the Figshare website (https://figshare.com/articles/dataset/brain_tumor_dataset/1512427/5). This dataset is a comprehensive collection of 3064 brain Magnetic Resonance (MR) images, derived from 233 distinct cases. These cases encompass three types of brain tumors: meningioma, pituitary, and glioma. The dataset is structured as follows.

− Meningioma Tumors: This category contains 708 MR images.
− Pituitary Tumors: This category is represented by 930 MR images.
− Glioma Tumors: This category is the largest, with 1426 MR images.

The images are stored in the “.mat” file format, which is compatible with MATLAB for easy implementation and analysis. Each image has a resolution of 512 × 512 pixels, providing a high level of detail for each case. The dataset has been divided into training and validation subsets. Approximately 80 % of the images (2451 images) are allocated for training purposes, while the remaining 20 % (613 images) are reserved for testing. The splitting process was conducted using the “splitEachLabel” toolbox from MATLAB, ensuring a random and unbiased division of the data.

**Fig. 6:**
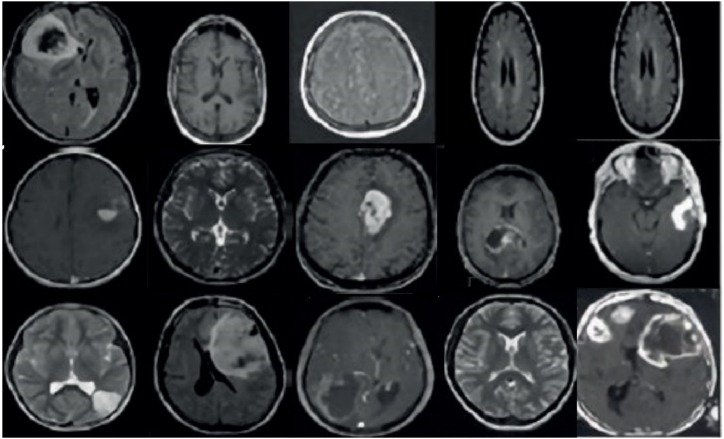
Several instances of datasets obtained from the Figshare dataset.

For further information and detailed explanations about the dataset, users are encouraged to visit the Figshare website. This dataset provides a valuable resource for researchers studying these types of brain tumors, offering a substantial volume of data for training and validating machine learning models. Fig. (6) showcases several instances of datasets obtained from the Figshare dataset.

### 7.2 System configuration

The proposed algorithm was evaluated on a computer with the following specifications. The system configuration features an Intel Core i7-8700K processor, a six-core processor with a base clock speed of 3.7 GHz and a maximum turbo frequency of 4.7 GHz, ideal for gaming and high-performance computing applications. It is equipped with 16 GB DDR4 RAM, the fourth generation of Double Data Rate (DDR) memory technology, providing improved performance and energy efficiency.

The NVIDIA GeForce GTX 1080 Ti graphics card, with 11 GB of GDDR5X memory and CUDA cores, is used for demanding tasks like gaming, 3D rendering, and machine learning. The system runs on Windows 10, a popular operating system developed by Microsoft, providing a powerful computing environment for evaluating the proposed algorithm. This configuration ensures effective testing and analysis of the algorithm, maximizing the system's capabilities.

### 7.3 Results and discussions

The generation of a confusion matrix, consisting of four distinct elements, is the final outcome of the binary classification process. Table 2 presents the confusion matrix pertaining to the binary classification of oral cancer, wherein a distinction is made between instances classified as malignant and those classified as non-cancerous. This classification scheme categorizes patients into two distinct groups. Table 2 presents the confusion matrix for the binary classification of brain tumors, specifically focusing on distinguishing between healthy and tumorous cases.

**Table 2:** Confusion matrix for binary classification of brain tumor.

Predicted	Actual	
	Tumorous	Healthy
Tumorous	True negative (TN)	False Positive (FP)
Healthy	False negative (FN)	True Negative (TN)

The Accuracy metric plays a significant role in evaluating the effectiveness of a classifier. The equation provided below represents a metric that assesses the accuracy level in data classification attained by a specific classification model. The classification accuracy is measured on a continuum ranging from 0 to 1, with a value of 1 indicating the highest level of accuracy and a value of 0 indicating the lowest level [equation (26)].

(26) $$ACC=\frac{TP+TN}{TP+TN+FP+FN}$$

The subsequent section presents the outcome of the simulation and provides a discourse on the utilization of the proposed MN-V2/CFO model for evaluating the efficacy of various techniques in brain tumor diagnosis. Table 3 showcases the outcomes of evaluating the minimum, maximum, and average accuracy values obtained from testing the proposed approach.

**Table 3:** Accuracy ratings for the proposed MN-V2/CFO model compared with other algorithm.

Number of Iteration	Accuracy (%)		
	Minimum	Maximum	Average
10	29.53	33.48	30.22
20	37.59	55.93	46.18
40	34.92	45.30	41.38
80	36.03	56.33	38.15
120	36.84	64.49	37.46
160	43.37	77.83	66.22
200	46.96	97.32	79.67

**Fig. 7:**
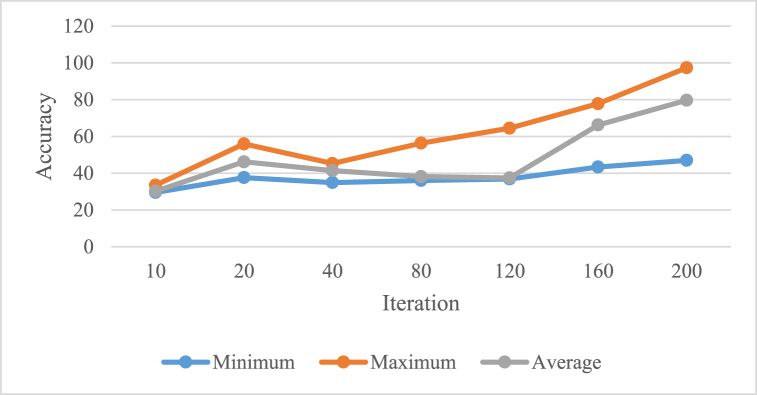
Convergence pattern of the proposed technique.

Based on the given table, the accuracy ratings for the proposed MN-V2/CFO model are compared with other algorithms at different iterations. It can be observed that the accuracy improves as the number of iterations increases. Overall, for the minimum, maximum, and average accuracy, there is an improvement in performance as the number of iterations increases. For example, at 10 iterations, the accuracy ranges from 29.53 % to 33.48 %, with an average accuracy of 30.22 %. However, when the number of iterations reaches 200, the accuracy ranges from 46.96 % to 97.32 %, with an average accuracy of 79.67 %. Additionally, fluctuations in accuracy can be observed at specific iteration numbers. For instance, at 160 iterations, the lowest accuracy is 43.37 % and the highest accuracy is 77.83 %, indicating significant performance variations in certain cases. In conclusion, the proposed MN-V2/CFO model demonstrates good accuracy performance at different iterations, with noticeable improvements as the number of iterations increases. However, further analysis and examination of other factors are necessary for a comprehensive evaluation of the model's performance compared to other algorithms. The convergence pattern of the proposed technique is illustrated in Fig. (7), depicting its performance across multiple iterations.

As can be observed from Fig. (7), the convergence pattern provides insights into how quickly or efficiently the technique reaches a stable or optimal solution. Analyzing the figure can help evaluate the effectiveness and performance of the proposed technique. Further examination of the figure, such as identifying any plateaus or fluctuations in the convergence pattern, can offer valuable information for improving the technique or understanding its limitations.

In the following, we present a detailed comparison analysis between the proposed method and some other state-of-the-art techniques in the field. The proposed method is evaluated in terms of its performance, advantages, and limitations when compared to existing approaches. The compared methods include Residual Network (RN) [35], wavelet transform and deep learning (WT/DL) [36], customized VGG19 [37], and Convolutional neural network (CNN) [38].

The objective is to assess the performance, strengths, and limitations of the proposed method in relation to these existing approaches. The RN has gained attention for its efficient training of deep neural networks using residual connections, but it may face difficulties in training and optimization due to its deep architecture and computational resources. WT/DL combines wavelet transform with deep learning to extract features from images but may have limitations in handling complex spatial relationships. Customized VGG19 modifies the VGG19 network architecture for specific image processing tasks, providing a deeper network with increased capacity for learning intricate visual patterns.

**Fig. 8:**
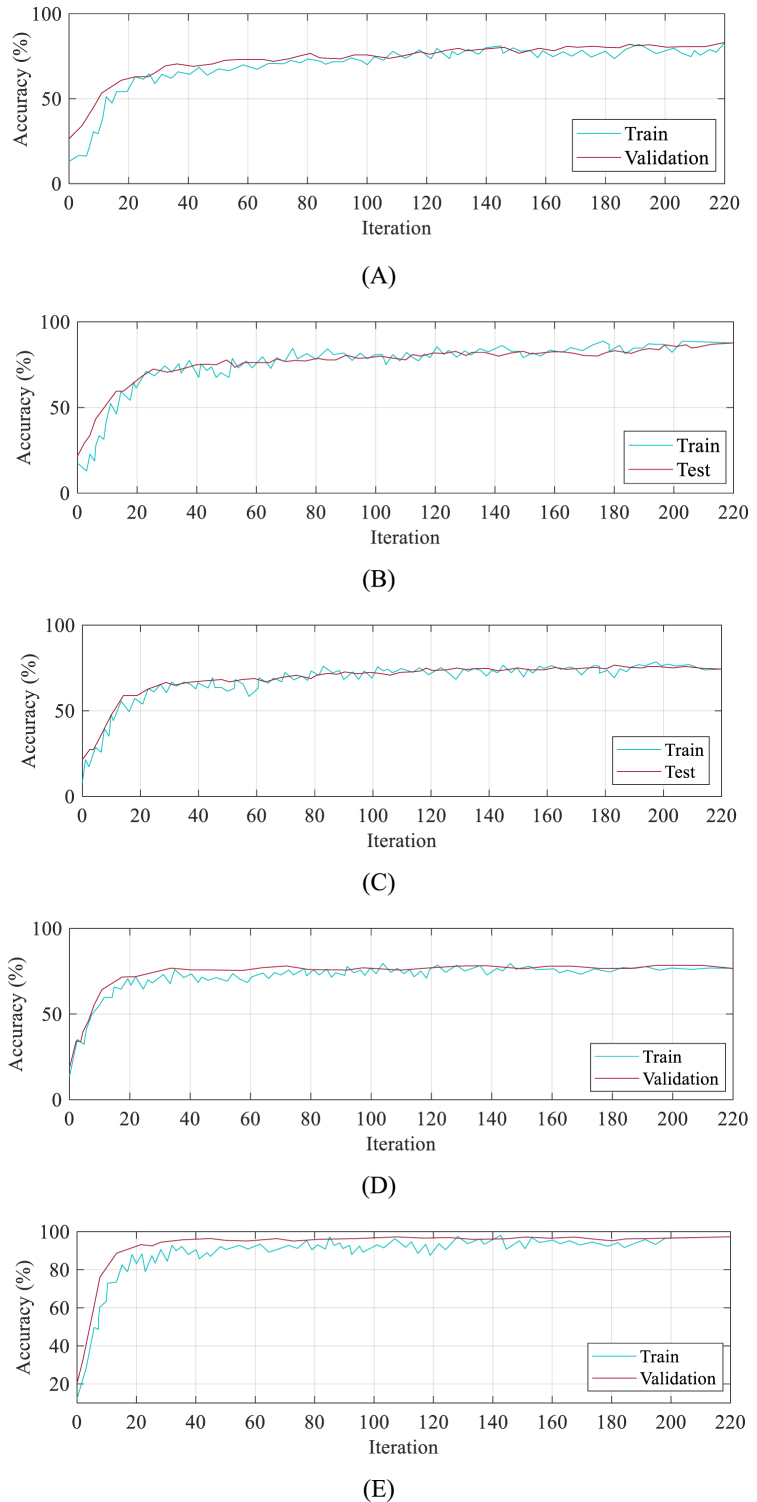
Training and Testing profile for: (A) RN (B) WT/DL (C) VGG19 (D) CNN, and (E) MN-V2/CFO.

However, the customization process may lead to a more complex training procedure and potential overfitting issues. Finally, Convolutional Neural Networks (CNNs) are widely used in image classification tasks due to their ability to learn hierarchical features from data. However, CNNs may suffer from limited receptive fields and struggle with capturing long-range dependencies in images. Fig. (8) shows the training and validation profiles for: (A) RN (B) WT/DL (C) VGG19 (D) CNN, and (E) MN-V2/CFO.

Fig. (8) enables analysis of crucial factors such as convergence speed, overfitting, and model stability. In the case of RN, the graph displays a gradual decrease in training error, albeit with slower convergence compared to other models. Nonetheless, the validation error also decreases, indicating successful generalization to unseen data. WT/DL exhibits rapid convergence in both training and validation errors, suggesting efficient learning, while the small gap between the curves indicates good generalization capabilities. VGG19 demonstrates consistent learning through a steady decline in training error, but there is a notable gap between the training and validation errors, suggesting potential overfitting or limited generalization.

CNN shows fast initial convergence followed by a slight increase in test error, indicating possible overfitting and reduced performance on unseen samples. On the other hand, MN-V2/CFO displays a smooth decrease in both training and validation errors, signifying effective learning and generalization, with a small gap between the curves denoting stable performance across different datasets. Analyzing these training and testing profiles helps identify the strengths and weaknesses of each model, providing valuable guidance for further analysis, model training, architecture design, and generalization techniques [equations (27), (28), (29), (30)].

(27) $$Accuracy=\frac{TP+TN}{TP+TN+FP+FN}$$

(28) $$Sensitivity=\frac{TP}{TP+FN}$$

(29) $$Precision=\frac{TP}{TP+FP}$$

(30) $$F1=\frac{2\times Senc\times Prc}{Senc+Prc}$$

Table 4 presents a comparison of the recommended strategy with other tactics on the “Figshare dataset” in terms of the F1-score, precision, accuracy, and sensitivity of the classification. The findings of this comparison highlight the performance differences among the different strategies [Table 4].

**Table 4:** Comparison of the recommended strategy with other tactics on the “Figshare dataset”.

Model	Precision	F1-score	Sensitivity	Accuracy
RN [35]	78.94	74.18	76.89	83.09
WT/DL [36]	84.85	77.34	76.24	87.69
VGG19 [37]	71.21	67.81	61.45	74.36
CNN [38]	76.50	68.01	64.91	76.65
MN-V2/CFO	97.68	86.22	80.12	97.32

The provided Table 4 compares the recommended strategy with other tactics on the "Figshare dataset" based on their performance metrics including precision, F1-score, sensitivity, and accuracy of the classification. Each model's scores are listed in the table for easy comparison. The results indicate that the recommended strategy, MN-V2/CFO, outperforms the other tactics across all the metrics. With a precision of 97.68 %, it achieves the highest level of accurate positive predictions. The F1-score, which considers both precision and recall, is also impressive at 86.22 %, indicating a good balance between precision and sensitivity.

In terms of sensitivity, the MN-V2/CFO model scores 80.12 %, meaning it effectively identifies a high percentage of true positives. Additionally, the accuracy of the MN-V2/CFO model is 97.32 %, reflecting its ability to correctly classify both positive and negative instances. In comparison, the other models demonstrate varying levels of performance. For example, the WT/DL model shows relatively high precision (84.85 %) and F1-score (77.34 %), suggesting good overall performance. However, its sensitivity (76.24 %) is slightly lower, indicating potential room for improvement in correctly identifying true positives. The RN model performs moderately well with precision, F1-score, sensitivity, and accuracy scores of 78.94 %, 74.18 %, 76.89 %, and 83.09 % respectively.

While these scores are decent, they are still lower than those achieved by the recommended MN-V2/CFO strategy. In contrast, the VGG19 and CNN models exhibit lower performance across all metrics compared to the recommended strategy. Both models show lower precision, F1-score, sensitivity, and accuracy, suggesting that they may struggle to accurately classify instances on the Figshare dataset. In conclusion, the findings from this comparison highlight the superior performance of the recommended MN-V2/CFO strategy on the Figshare dataset. However, further analysis and experimentation may be required to determine the suitability of these models for specific tasks or datasets.

### 7.4 Advantages of the proposed MN-V2/CFO strategy

The proposed method exhibits several advantages over the compared techniques. It combines the efficiency of RN, the multi-resolution analysis capability of WT/DL, the capacity to learn intricate patterns from Customized VGG19, and the ability to capture long-range dependencies from CNN. Moreover, it overcomes its limitations by offering improved training and optimization, handling complex spatial relationships, and capturing long-range dependencies simultaneously.

### 7.5 Limitation of study

For the proposed model, the following limitations should be considered.

− Dataset Limitations: The performance and generalizability of the proposed model heavily rely on the quality, diversity, and representativeness of the dataset used for training and evaluation. If the dataset is limited in size or lacks diversity in terms of tumor types, patient demographics, or imaging protocols, the model's performance may be affected. Additionally, the dataset used in the study, such as the Figshare dataset, may have its own limitations, including potential biases or inconsistencies in data collection.
− Generalizability: The proposed model's performance should be validated on external datasets to assess its generalizability across different populations, imaging devices, and clinical settings. Without such validation, the model's effectiveness in real-world scenarios may be uncertain.
− Ethical Considerations: The study should adhere to ethical guidelines and ensure the privacy and confidentiality of patient data. It is crucial to obtain appropriate informed consent and comply with relevant regulations to protect patient rights and privacy.
− Interpretability: Deep learning models, including Mobilenetv2, are often considered black-box models, making it challenging to interpret the decision-making process. Understanding the rationale behind the model's predictions and identifying potential sources of bias or errors may be difficult.
− Computational Resources: The proposed model may require significant computational resources, including processing power and memory, to train and deploy. This could limit its practical implementation in resource-constrained environments or hinder its scalability.
− Clinical Validation: While the study demonstrates the potential of the proposed model in improving brain tumor diagnosis, further clinical validation is necessary to assess its impact on patient outcomes, treatment decisions, and healthcare workflows. Collaborations with medical professionals and rigorous clinical trials are essential to evaluate the model's real-world effectiveness.

### 7.6 Clinical significance of your findings

The clinical significance of the findings presented in this study is substantial. The proposed model, which combines the Mobilenetv2 deep learning model with the Contracted Fox Optimization Algorithm (CFOA), demonstrates improved accuracy and efficiency in brain tumor diagnosis using MRI scans. This has several important clinical implications.

Firstly, the improved accuracy of the proposed model can lead to more precise and reliable identification and characterization of brain tumors. Accurate diagnosis is crucial for determining the appropriate treatment strategies and interventions for patients. By reducing false positives and false negatives, the proposed model can help clinicians make more informed decisions and avoid unnecessary treatments or delays in necessary interventions.

Secondly, accurate and efficient brain tumor diagnosis is essential for developing effective treatment plans. The proposed model's ability to accurately identify and characterize brain tumors can aid in determining the optimal treatment approach, such as surgery, radiation therapy, or chemotherapy. This can lead to more targeted and personalized treatment strategies, potentially improving patient outcomes and reducing the burden of unnecessary treatments.

Additionally, the enhanced efficiency of the proposed model can significantly reduce the time and resources required for brain tumor diagnosis. Traditional methods of diagnosis often involve manual interpretation of MRI scans by radiologists, which can be time-consuming and subject to human error. By automating the diagnosis process, the proposed model can expedite the detection and classification of brain tumors, allowing for faster treatment planning and initiation.

Moreover, the proposed model's accuracy and efficiency make it a promising tool for telemedicine and remote diagnosis. With the increasing availability of telehealth services, the ability to accurately diagnose brain tumors remotely can improve access to specialized care for patients in underserved areas or those who face geographical barriers. The proposed model can facilitate remote consultations and enable timely diagnosis and treatment recommendations, ultimately improving patient outcomes and reducing healthcare disparities.

In general, the combination of improved precision, personalized treatment strategies, time and resource efficiency, and potential for telemedicine makes the findings of this study highly significant in the field of brain tumor diagnosis. The proposed model has the potential to revolutionize the way brain tumors are diagnosed and treated, ultimately benefiting patients and healthcare systems alike.

## 8 Conclusions

Brain tumors are cancerous formations characterized by the aberrant proliferation of cells within the cerebral region, which can exert profound deleterious effects on an individual's physiological state and overall quality of life. The timely and precise identification of brain tumors is of utmost importance in order to facilitate optimal therapeutic interventions and enhance patient prognoses. Medical imaging modalities, such as magnetic resonance imaging (MRI), serve a crucial function in the identification and characterization of brain tumors. Nevertheless, the process of diagnosing brain tumors via MRI scans can present significant challenges. The process of human interpretation of these scans is characterized by subjectivity, a significant time investment, and susceptibility to errors. Furthermore, the efficacy of the process is heavily contingent upon the proficiency and knowledge of radiologists, whose accessibility and consistency may not be universally guaranteed. In order to tackle these challenges, scholars have resorted to the utilization of artificial intelligence (AI) and machine learning methodologies. Deep learning, which is a subfield of AI, has demonstrated considerable potential in the analysis of medical imaging. By employing deep learning models on extensive datasets, these models can acquire the ability to discern intricate patterns and characteristics within medical images, thereby facilitating precise diagnostic procedures. Optimization algorithms serve as a valuable complement to deep learning techniques, as they contribute to enhancing the performance and generalizability of models. Therefore, the present study demonstrated the potential of utilizing deep learning and optimization techniques in the field of medical imaging, specifically focusing on brain tumor diagnosis. This innovative methodology addressed the challenges involved in identifying and characterizing brain tumors using MRI scans. By implementing Mobilenetv2, a robust deep learning model, optimized by the Contracted Fox Optimization Algorithm, a new approach was introduced to enhance the precision of tumor detection. The Contracted Fox Optimization Algorithm (CFOA), a metaheuristic algorithm, efficiently optimized the hyperparameters of Mobilenetv2. This optimization process ensured that the model effectively learnt and extracted meaningful features from MRI scans, resulting in improved diagnostic performance. The adoption of this automated methodology significantly improved the accuracy of brain tumor diagnosis. It also reduced reliance on human interpretation, which was prone to subjective biases and errors. This automated approach not only enhanced diagnostic precision but also expedited the diagnosis process, enabling timely identification and treatment of brain tumors. The successful implementation of the model on the Figshare dataset, which encompassed a comprehensive collection of MRI scans, further validated its effectiveness and potential for real-world application. More clarification about the efficiency of the study was addressed by comparing its results with some state-of-the-art methods, including Network (RN), wavelet transform and deep learning (WT/DL), customized VGG19, and Convolutional neural network (CNN). Simulation results highlighted the superiority of the proposed model compared to other tactics. The recommended strategy achieves a precision of 97.68 %, an F1-score of 86.22 %, a sensitivity of 80.12 %, and an accuracy of 97.32 %. However, further research was required to refine the model and explored its applicability in diverse clinical settings. These future investigations could aim to optimize the model's performance across various patient populations and MRI acquisition protocols.

## Additional information

No additional information is available for this paper.

## CRediT authorship contribution statement

**Lu Xu:** Writing – review & editing, Software, Resources, Formal analysis, Data curation. **Morteza Mohammadi:** Writing – review & editing, Writing – original draft, Methodology, Data curation, Conceptualization.

## Declaration of competing interest

The authors declare that they have no known competing financial interests or personal relationships that could have appeared to influence the work reported in this paper.
